# Assessment of occult cervical metastases in oral cavity squamous cell carcinoma: Findings from a tertiary-level hospital study

**DOI:** 10.4317/jced.63221

**Published:** 2025-10-17

**Authors:** Clara López-Martínez, Javier González-Martín-Moro, Daniel López-Martínez, Alba García-López-Chicharro, Marta María Pampín-Martínez, José Luis Cebrián-Carretero

**Affiliations:** 1 Oral and Maxillofacial Surgery Department, University Hospital La Paz. IdiPAZ. Madrid, Spain; 2 School of Computer Science Engineering, Universidad of Valladolid. Valladolid, Spain

## Abstract

**Background:**

Cervical lymph node metastasis is the key prognostic factor in head and neck squamous cell carcinoma (HNSCC), particularly in oral cavity squamous cell carcinoma (OCSCC). Detecting occult metastases in clinically node-negative (cN0) necks remains challenging, as CT and PET-CT exhibit limited sensitivity and specificity. This study aimed to assess the incidence of occult cervical metastases in OCSCC patients, evaluate imaging accuracy, and identify associated histopathological factors.

**Material and Methods:**

We retrospectively analyzed 43 OCSCC patients undergoing elective neck dissection (END) from 2017 - 2024 at a tertiary hospital. Data on clinical staging, imaging findings, histopathological characteristics (depth of invasion [DOI], lymphovascular invasion [LVI]) and tumor location, were collected. Statistical tests (t-tests, chi-square) were performed using R v3.6.1.

**Results:**

Occult cervical metastases occurred in 23.26% of patients (10/43). CT sensitivity was 70.27%, specificity 82.86%; PET-CT showed 81.25% sensitivity and 71.43% specificity. Patients with occult metastases had a higher mean DOI (13.13 ± 10.39 mm) compared to those without (8.1 ± 5.85 mm), though this difference was not statistically significant (p=0.172). LVI was absent in the metastatic group but present in 9.09% of non-metastatic cases. Tumor location did not significantly correlate with occult metastases (p=0.801).

**Conclusions:**

Our findings confirm DOI as a crucial predictor of occult metastases in OCSCC, reinforcing the importance of END in high-risk patients. While PET-CT showed higher sensitivity than CT, both imaging modalities had limitations in detecting micrometastases. Further studies with larger sample sizes are needed to validate these findings and explore additional predictive factors, potentially integrating machine learning models for improved risk stratification.

## Introduction

Head and neck cancers represent the seventh most common cancer worldwide (1). In the absence of detectable distant metastases, the presence of cervical lymph node metastases stands out as the single most significant independent adverse prognostic factor influencing disease-free and overall survivals in patients with head and neck squamous cell carcinoma (HNSCC). Factor such as the number of lymph nodes and the level(s) in the neck, the size of metastases, and the presence of extracapsular spread or soft tissue deposits have all been identified as important indicators of prognosis in HNSCC patients (2). Thus, accurately assessing the status of cervical lymph nodes is pivotal for the selection of an adequate therapeutic decision. Originally, the assessment of cervical metastasis in HNSCC relied solely on clinical examination (palpable, indurated, and/or fixed adenopathies). However, studies have reported the presence of hidden cervical metastases (3). Currently, contrast-enhanced computed tomography (CT) and magnetic resonance imaging serve as standard non-invasive imaging modalities for detecting regional nodal involvement in HNSCC patients. Yet, both exhibit limitations in distinguishing between metastatic and nonmetastatic lymph nodes, with CT showing 77% sensitivity and MRI 72%, while their specificities are comparable (85% and 84%, respectively) (4). MRI excels in assessing local soft tissue tumoral extension. However, to limit the number of complementary examinations, only one of the two forms of imaging should be performed (3). Imaging criteria for evaluation of metastatic cervical lymph nodes in HNSCC are summarized in Table 1 (5).

Table 1

Functional imaging, particularly 18F-FDG PET-CT, boasts a sensitivity ranging from 67% to 96% in detecting cervical metastasis, and a specificity between 82% to 100%. Several studies have investigated the utility of 18F-FDG PET-CT in populations initially classified as N0 either clinically or through CT/MRI scans. It has shown a superior sensitivity in uncovering occult cervical metastases. Consequently, PET-CT is now preferred for evaluating distant spread and synchronous tumors. The active management for metastatic neck disease in HNSCC includes elective neck dissection or elective neck irradiation. Two primary arguments support surgical intervention for treating neck metastasis. Firstly, neck dissection aids in accurately staging the disease, thereby guiding decisions regarding additional treatments. Secondly, it enhances locoregional control. But the real area of controversy is the management of clinically node-negative (cN0) neck. The overall risk of occult metastasis in cN0 necks ranges from 10% to 30% depending on the tumor characteristics (6). The management of the cN0 neck in early-stage HNSCC remains controversial, with several strategies available, including END, sentinel lymph node biopsy (SLNB), and observation with therapeutic neck dissection (TND) upon nodal recurrence. END demonstrates improved overall survival (OS) and disease-free survival (DFS) versus observation, particularly in patients with a higher risk of occult metastasis. However, END can lead to unnecessary surgical morbidity in patients without nodal involvement. SLNB, a less invasive alternative, allows for selective neck dissection only in cases with positive sentinel nodes. SLNB has shown comparable outcomes to END in terms of OS, DFS, and nodal recurrence, with the added benefit of reduced morbidity and cost, though its adoption is limited by technical complexity and lack of widespread standardization. Observation, or a "watchful waiting" approach, involves close clinical and radiological follow-up, with TND performed only if nodal recurrence is detected. While this strategy avoids unnecessary surgery in patients without metastasis, it carries the risk of delayed treatment and poorer outcomes if recurrence is detected at an advanced stage. Recent network meta-analyses suggest that END remains the most effective strategy for maximizing survival and reducing nodal recurrence, though SLNB shows promise as a non-inferior alternative, particularly in early-stage OSCC with low tumor depth of invasion (DOI). Further research is needed to compare the long-term outcomes, morbidity, and quality of life associated with these strategies (7 - 11). In this paper, our focus will be directed towards oral cavity squamous cell carcinoma, where cervical lymph node metastases critically impact prognosis. Treatment of patients with early OCSCC without clinical nodal disease is a subject of ongoing debate. The decision to perform END in patients with cT1-2 N0 OCSCC is commonly based on tumor thickness or depth of invasion (DOI), which has consistently demonstrated an association with risk of occult nodal metastasis (12 , 13). This study aims to evaluate the incidence of occult cervical metastases in OCSCC patients treated at La Paz University Hospital, assess the diagnostic accuracy of different imaging modalities for detecting cervical metastases, and analyze histopathological factors associated with higher rates of occult metastases.

## Material and Methods

**Figure 1:**
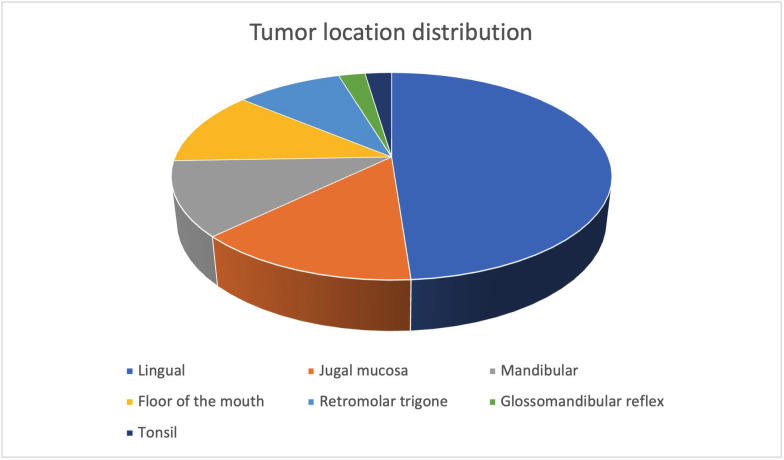
Tumor location distribution.

- Patient demographics and data collection In this retrospective study, the guidelines of the Helsinki Declaration are followed. Patients with history of OCSCC who underwent neck dissection between January 2017 and January 2024 at the Department of Oral and Maxillofacial Surgery at La Paz University Hospital were recruited. Patients were enrolled if the following criteria were met: (a) patients with OCSCC diagnosed by biopsy; (b) patients with cN0 status; (c) patients with curative intended surgery; (d) patients undergoing resection of the primary tumor with END; (e) patient with at least one preoperative imaging test performed (CT, MR or PET). Data on clinical nodal staging, imaging findings (CT and 18F-FDG PET-CT), and pathological nodal staging were collected from patient records. Histopathological characteristics including tumor DOI, perineural and lymphovascular invasion, tumor size, pT, pN and location were also recorded (Fig 1.)

1

All preoperative clinical and postoperative histological tumor staging of cases were recorded according to the 8th edition of the AJCC TNM classification. A thorough preoperative physical examination together with CT or 18F-FDG PET-CT was performed on patients with a clear pathological diagnosis or suspected as OCSCC. - Statistical analysis All statistical analyses were performed using R version 3.6.1 (R Foundation for Statistical Computing, Vienna, Austria). Continuous variables were expressed as mean ± standard deviation, while categorical variables were presented as frequencies and percentages. To compare differences between the occult metastasis group and the non-lymph node metastasis group, appropriate statistical tests were applied. Specifically, t-tests were used for continuous variables, and chi-square tests or Fisher's exact tests were used for categorical variables, depending on the distribution and sample size. A p-value of less than 0.05 was considered statistically significant. All analyses were conducted with the aim of identifying factors associated with occult cervical metastases in patients with OCSCC.

## Results

The study included 43 patients diagnosed with OCSCC who underwent END between January 2017 and January 2024. The mean age of the patients was 62.1 ± 13.5 years. Among these patients, histopathology revealed occult cervical metastases in 10 cases (23.26%), while 33 (76.74%) showed no nodal involvement. This demonstrates that approximately 25% of clinically node-negative (cN0) patients harbored hidden metastases. - Accuracy of Imaging Modalities: The accuracy of preoperative imaging modalities in detecting occult metastases was evaluated. CT showed 70.27% sensitivity and 82.86% specificity, while PET-CT demonstrated 81.25% sensitivity and 71.43% specificity. Although PET-CT showed higher sensitivity than CT, both modalities had limitations in accurately identifying occult metastases, particularly in patients with cN0 necks (Table 2).

Table 2

- Clinicopathological Factors and Occult Metastases: The relationship between various clinicopathological factors and the presence of occult metastases was analyzed. Patients with occult metastases were older (mean age 65.97 ± 12.28 years vs. 59.3 ± 15.65 years, p=0.239) and had greater depth of invasion (13.13 ± 10.39 mm vs. 8.1 ± 5.85 mm, p=0.172), though neither difference reached statistical significance. Perineural invasion was more commonly observed in patients with occult metastases, although the small sample size limited the ability to detect significant differences. Lymphovascular invasion (LVI) was observed in 3 patients (9.09%) in the non-metastatic group, while none of the patients with occult metastases showed LVI (p=1). Tumor size did not differ significantly between groups (22.9 ± 12.35 mm vs. 22.88 ± 11.12 mm, p=0.996) (Table 3).

Table 3

- Location and Occult Metastases: The location of the primary tumor was also analyzed in relation to the presence of occult metastases. The most common tumor location was the lingual region (metastatic: 50%, non-metastatic: 48.48%), followed by the mandibular region (metastatic: 20%, non-metastatic: 9.09%) and retromolar trigone (metastatic: 10%, non-metastatic: 9.09%). Tonsillar involvement was rare (non-metastatic: 3.03%, metastatic: 0%). There was no significant association between tumor location and the presence of occult metastases (p=0.801) (Table 4).

Table 4

## Discussion

The current, 8th edition of the American Joint Committee on Cancer (AJCC) guidelines utilizes DOI as a key parameter in tumor classification (T) with oral cavity T1 and T2 being 5 mm or 10 mm, respectively (14). Prior to the modification of the AJCC guidelines, multiple studies identified DOI as a more reliable predictor of occult lymph node metastasis in OCSCC compared to other metrics. Crissman et al. (1984) determined that invasive tumors with large, cohesive aggregates had a more favorable prognosis than those composed of thin, irregular, individual cells, reinforcing the relevance of DOI (15). Kane et al. (2006) further confirmed that, in early-stage OCSCC, DOI is the most critical histopathological indicator of subclinical neck disease, with tumors exhibiting a DOI of 5 mm or more being at a high risk for nodal metastasis (16). In future studies, it would be valuable to incorporate the Worst Pattern of Invasion (WPOI) as a risk factor, particularly WPOI-5, which has been shown to be a significant predictor of occult cervical metastases in early-stage OCSCC. WPOI-5, characterized by dispersed tumor satellites 1 mm from the main tumor mass, is strongly associated with occult metastases, even in cases with a DOI &lt; 4 mm, suggesting its critical role in guiding surgical decisions such END. Intraoperative identification of WPOI-5 has demonstrated high accuracy, specificity, and sensitivity, making it a valuable tool for improving risk stratification and reducing overtreatment in early-stage OCSCC (17). The findings of this study highlight the challenges in accurately predicting the presence of occult cervical metastases in patients with OCSCC. Despite advances in imaging modalities, a significant proportion of patients with cN0 necks harbor occult metastases, as evidenced by the 23.26% incidence rate in our cohort. This is consistent with the existing literature and underscores the importance of END in the management of early-stage OCSCC, particularly in cases with high-risk features such as increased DOI (6 , 10 , 11 , 16). DOI has been consistently identified as a critical factor associated with the risk of occult nodal metastasis. In our study, patients with occult metastases had a higher mean DOI compared to those without metastases, although this difference did not reach statistical significance. This may be due to the relatively small sample size, which limits the statistical power to detect significant differences. Nonetheless, the trend aligns with previous studies that have demonstrated a strong correlation between DOI and the likelihood of occult metastases (10 , 12 , 16). Interestingly, lymphovascular invasion was absent in patients with occult metastases but present in 9.09% of patients without metastases. This finding is somewhat counterintuitive, as LVI is generally considered a poor prognostic factor associated with increased metastatic potential (18). However, the absence of LVI in the metastatic group may be attributed to the small sample size or the possibility that other factors, such as DOI, play a more significant role in the dissemination of tumor cells to regional lymph nodes. Primary tumor location showed no significant correlation with occult metastases in our study. The lingual region was the most common site of involvement in both metastatic and non-metastatic groups, followed by the mandible and retromolar trigone. This suggests that tumor location may not be a reliable predictor of occult metastases, and other factors (DOI and tumor size) should be given greater consideration in clinical decision-making (19 , 20). In terms of imaging accuracy, PET-CT demonstrated superior sensitivity (81.25%) compared to CT (70.27%) in detecting occult metastases. However, both modalities showed limited efficacy in identifying micrometastases in cN0 necks (21 , 22). This reinforces the need for complementary diagnostic approaches, including histopathological evaluation, for precise staging and therapeutic guidance. Future studies should explore the potential of machine learning models to improve the identification of patients at higher risk of occult cervical metastases in early-stage OSCC. As demonstrated by Bur et al. (2019), machine learning algorithms, particularly decision forest models, outperformed traditional DOI methods in predicting occult nodal disease, offering higher sensitivity and specificity. These models could help refine clinical decision-making by reducing unnecessary neck dissections while ensuring that patients with occult metastases receive timely and appropriate treatment (23). Further development of these algorithms, using high-quality, multi-institutional data, could enhance their predictive accuracy and clinical utility, paving the way for more personalized and effective management strategies in early-stage OSCC.

## Conclusions

In conclusion, our study reinforces the importance of DOI as a key factor in predicting occult cervical metastases in patients with early-stage OCSCC. While CT and PET-CT are valuable tools for initial staging, they may not be sufficient to detect micrometastases in cN0 necks. For patients with cN0 necks at risk of micrometastases, observation alone is not a viable option, and at a minimum, SLNB should be considered to accurately assess nodal status. END remains a crucial intervention for accurate staging and improving locoregional control in high-risk patients. Further studies with larger sample sizes are needed to validate these findings and to explore additional histopathological factors that may influence the risk of occult metastases. Additionally, the integration of machine learning models could play a pivotal role in more accurately predicting occult nodal metastasis, potentially reducing unnecessary surgeries and better identifying patients at the highest risk of hidden metastasis.

## Figures and Tables

**Table 1:** Imaging Criteria for Evaluation of Metastatic Cervical Lymph Nodes in HNSCC.

Size (individual, nonclustered nodes)	Maximal axial diameter, abnormal if > 15 mm for level I or level II nodes; > 8 mm for retropharyngeal nodes; and > 10 mm for all other nodesMinimum axial diameter, abnormal if > 11 mm for level II nodes; > 5 mm for retropharyngeal nodes; and > 10 mm for all other nodes
Internal abnormality	Also referred to as nodal inhomogeneity or central necrosisRadiologically detectable when areas are > 3 mm in sizeCystic change: thin (< 2 mm) enhancing capsule, homogeneous fluid content (> 70% with HU < 20 on CT); may be a distinct imaging and pathologic entity from central necrosis (which have thicker solid walls and complex central low attenuation)
Margins	ENE suspected if:Enhancing, thickened nodal rimIrregular or poorly defined nodal marginsInfiltration of the adjacent fat planes or invasion to adjacent structures
Shape	Abnormal rounded appearance with loss of the normal ovoid shapeL/T ratio (longitudinal length/ transaxial width): ratio < 2 suggestive of metastasis; ratio > 2 suggestive of reactive node (round/ sphere shape vs lima bean shape)
Grouping	Cluster or group of 3 or more nodes in the primary drainage level of the tumor siteMaximal axial diameter, abnormal if 8-15 mmMinimum axial diameter, abnormal if 9-10 mm for level II nodes; 8-9 mm for all other nodes

1

**Table 2:** Accuracy of Imaging Modalities.

	Sensitivity	Specificity
CT	70.27%	82.86%
18F-FDG PET-CT	81.25%	71.43%

2

**Table 3:** Univariate Analysis of Potential Clinicopathological Parameters Predictive of Occult Lymph Node Metastasis in OCSCC.

Variables	Occult Metastasis (n = 10)	No occult metastasis (n = 33)	Conf. Int.	P-value
Age	59.3 ± 15.65	65.97 ± 12.28	(-18.36, 5.02)	0.239
Sex	3 (30%)	13 (39.39%)	(0.094, 3.617)	0.719
DOI	13.13 ± 10.39	8.1 ± 5.85	(-2.57, 12.63)	0.172
PNI	4 (40%)	7 (21.21%)	(0.39, 14.03)	0.248
LVI	0	3 (9.09%)	(0, 8.26)	1
G1	2 (20%)	6 (18.18%)	-	1
G2	8 (80%)	25 (75.76%)		
G3	0	2 (6.06%)		
Size	22.9 ± 12.35	22.88 ± 11.12	(-9.35, 9.39)	0.996

3

**Table 4:** Analysis of Location as a Potential Predictive Factor of Occult Lymph Node Metastasis in OCSCC.

Variables	Occult Metastasis (n = 10)	No Occult Metastasis (n = 33)	P-value
Lingual	5 (50%)	16 (48.48%)	0.801
Jugal mucosa	2 (20%)	4 (12.12%)	
Mandibular	2 (20%)	3 (9.09%)	
Floor of the mouth	0	5 (15.15%)	
Retromolar trigone	1 (10%)	3 (9.09%)	
Glossomandibular reflex	0	1 (3.03%)	
Tonsil	0	1 (3.03%)	

4

## Data Availability

The datasets used and/or analyzed during the current study are available from the corresponding author.

## References

[1] Gormley M, Creaney G, Schache A, Ingarfield K, Conway DI (2022). Reviewing the epidemiology of head and neck cancer: definitions, trends and risk factors. Br Dent J.

[2] Ferlito A, Rinaldo A, Devaney KO, Hamakawa H (2008). Detection of lymph node micrometastases in patients with squamous carcinoma of the head and neck. Eur Arch Oto-Rhino-Laryngol Off J Eur Fed Oto-Rhino-Laryngol Soc EUFOS Affil Ger Soc Oto-Rhino-Laryngol - Head Neck Surg.

[3] Pauzie A, Gavid M, Dumollard JM, Timoshenko A, Peoc’h M, Prades JM (2016). Infracentimetric cervical lymph node metastasis in head and neck squamous cell carcinoma: Incidence and prognostic value. Eur Ann Otorhinolaryngol Head Neck Dis.

[4] Sun J, Li B, Li C, Li Y, Su F, Gao Q (2015). Computed tomography versus magnetic resonance imaging for diagnosing cervical lymph node metastasis of head and neck cancer: a systematic review and meta-analysis. OncoTargets Ther.

[5] Kelly HR, Curtin HD (2017). Chapter 2 Squamous Cell Carcinoma of the Head and Neck-Imaging Evaluation of Regional Lymph Nodes and Implications for Management. Semin Ultrasound CT MR.

[6] Majumdar KS, Rao VUS, Prasad R, Ramaswamy V, Sinha P, Subash A (2020). Incidence of Micrometastasis and Isolated Tumour Cells in Clinicopathologically Node-Negative Head and Neck Squamous Cell Carcinoma. J Maxillofac Oral Surg.

[7] Hanai N, Asakage T, Kiyota N, Homma A, Hayashi R (2019). Controversies in relation to neck management in N0 early oral tongue cancer. Jpn J Clin Oncol.

[8] Al-Moraissi EA, Alkhutari AS, de Bree R, Kaur A, Al-Tairi NH, Pérez-Sayáns M (2024). Management of clinically node-negative early-stage oral cancer: network meta-analysis of randomized clinical trials. Int J Oral Maxillofac Surg.

[9] Wu JX, Hanson M, Shaha AR (2019). Sentinel node biopsy for cancer of the oral cavity. J Surg Oncol.

[10] Jang SS, Davis ME, Vera DR, Lai SY, Guo TW (2023). Role of sentinel lymph node biopsy for oral squamous cell carcinoma: current evidence and future challenges. Head Neck.

[11] D’Cruz AK, Vaish R, Kapre N, Dandekar M, Gupta S, Hawaldar R (2015). Elective versus Therapeutic Neck Dissection in Node-Negative Oral Cancer. N Engl J Med.

[12] Aaboubout Y, van der Toom QM, de Ridder MAJ, De Herdt MJ, van der Steen B, van Lanschot CGF (2021). Is the Depth of Invasion a Marker for Elective Neck Dissection in Early Oral Squamous Cell Carcinoma?. Front Oncol.

[13] Chen T, Terng S, Lee L, Lee S, Ng S, Kang C (2024). Is elective neck dissection justified in cT2N0M0 oral cavity cancer defined according to the AJCC eighth edition staging system?. Cancer Med.

[14] Lydiatt WM, Patel SG, O’Sullivan B, Brandwein MS, Ridge JA, Migliacci JC (2017). Head and Neck cancers-major changes in the American Joint Committee on cancer eighth edition cancer staging manual. CA Cancer J Clin.

[15] Crissman JD, Liu WY, Gluckman JL, Cummings G (1984). Prognostic value of histopathologic parameters in squamous cell carcinoma of the oropharynx. Cancer.

[16] Kane SV, Gupta M, Kakade AC, D’ Cruz A (2006). Depth of invasion is the most significant histological predictor of subclinical cervical lymph node metastasis in early squamous carcinomas of the oral cavity. Eur J Surg Oncol J Eur Soc Surg Oncol Br Assoc Surg Oncol.

[17] Beute JE, Greenberg LA, Wein LE, Kapustin DA, Fan J, Dowling EM (2023). WPOI-5: Accurately Identified at Intraoperative Consultation and Predictive of Occult Cervical Metastases. Head Neck Pathol.

[18] Arora A, Husain N, Bansal A, Neyaz A, Jaiswal R, Jain K (2017). Development of a New Outcome Prediction Model in Early-stage Squamous Cell Carcinoma of the Oral Cavity Based on Histopathologic Parameters With Multivariate Analysis: The Aditi-Nuzhat Lymph-node Prediction Score (ANLPS) System. Am J Surg Pathol.

[19] Farrokhian N, Holcomb AJ, Dimon E, Karadaghy O, Ward C, Whiteford E (2022). Development and Validation of Machine Learning Models for Predicting Occult Nodal Metastasis in Early-Stage Oral Cavity Squamous Cell Carcinoma. JAMA Netw Open.

[20] Jin W, Zhu M, Zheng Y, Wu Y, Ding X, Wu H (2022). Perineural invasion, lactate dehydrogenase, globulin, and serum sodium predicting occult metastasis in oral cancer. Oral Dis.

[21] Lee HJ, Kim J, Woo HY, Kang WJ, Lee JH, Koh YW (2015). 18F-FDG PET-CT as a supplement to CT/MRI for detection of nodal metastasis in hypopharyngeal SCC with palpably negative neck. The Laryngoscope.

[22] Burian E, Palla B, Callahan N, Pyka T, Wolff C, von Schacky CE (2022). Comparison of CT, MRI, and F-18 FDG PET/CT for initial N-staging of oral squamous cell carcinoma: a cost-effectiveness analysis. Eur J Nucl Med Mol Imaging.

[23] Bur AM, Holcomb A, Goodwin S, Woodroof J, Karadaghy O, Shnayder Y (2019). Machine learning to predict occult nodal metastasis in early oral squamous cell carcinoma. Oral Oncol.

